# Number of Children and Cognitive Aging in Older Americans: The Role of Socioeconomic Status and Gender Differences

**DOI:** 10.1093/geroni/igaf122.2566

**Published:** 2025-12-31

**Authors:** Dongfang Hong, Wuyi Dong

**Affiliations:** University of Massachusetts Boston, Boston, Massachusetts, United States; University of Massachusetts Boston, Boston, Massachusetts, United States

## Abstract

Evidence on the influence of the number of children on late-life cognitive functions remains inconsistent, particularly regarding its specific effects on men and women. This study analyzed data from the Health and Retirement Study (HRS) (1998–2016), a nationally representative longitudinal survey of older Americans. The sample included 28,391 individuals aged 55 and older. Cognitive function was assessed using a 27-point TICS score incorporating word recall, serial 7s, and backward counting. The number of children was categorized as none, one or two, and three or more. Linear mixed-effects models examined the association between the number of children and cognitive function, adjusting for sociodemographic, health, and lifestyle factors. In the unadjusted model, having three or more children was associated with a significant decline in cognitive functioning (β = -0.462, p < 0.001) compared to those with no children. However, after adjusting for covariates, this association reversed, with three or more children linked to higher cognitive function (β = 0.335, p < 0.01). Stepwise analyses indicated that the initial negative association was primarily explained away by socioeconomic status (SES), particularly education. Gender-stratified models revealed that the positive effect of having three or more children was stronger in men (β = 0.432, p < 0.05) than in women (β = 0.234, not significant). These findings underscore the role of SES in shaping the relationship between family structure and gender-specific cognitive trajectories. Future research should explore mechanisms such as economic stability and caregiving burden to better understand how parenthood influences late-life cognitive trajectories.

